# Accuracy of 64-multidetector computed tomography in diagnosis of adnexal tumors

**DOI:** 10.1186/1757-2215-4-15

**Published:** 2011-08-17

**Authors:** Fatemeh Gatreh-Samani, Mohammad Kazem Tarzamni, Elaheh Olad-Sahebmadarek, Ali Dastranj, Aimaz Afrough

**Affiliations:** 1 Women's Reproductive Health Research Center, Alzahra Hospital, Tabriz University (Medical Sciences), Tabriz, Eastern Azerbaijan, Iran; 2 Department of Radiology, Imam Reza Hospital, Tabriz University (Medical Sciences), Tabriz, Eastern Azerbaijan, Iran; 3 Department of Pathology, Alzahra Hospital, Tabriz University (Medical Sciences), Tabriz, Eastern Azerbaijan, Iran

**Keywords:** Adnexal diseases, diagnostic imaging, ovarian neoplasms, tomography, spiral computed

## Abstract

**Background:**

Adnexal cancers are in fifth place among the tumors with the highest mortality in the female population. The aim of the present study was to evaluate the accuracy of Multi-detector computed tomography (MDCT) on a 64-multislice CT scanner in the detection and differentiation of adnexal masses stages.

**Methods:**

During the present prospective study, 95 women with a primary diagnosis of ovarian mass in base of clinical examination and ultrasonographic findings underwent preoperative evaluation by a 64-slice MDCT with a section thickness of 0.6 mm, 50% overlap and reconstructed images. Afterward, results of MDCT were compared with surgical and histopathological findings, and the sensitivity, specificity, positive and negative predictive value and accuracy were determined.

**Results:**

The mean age of patients was 48.63 ± 13.93 years. MDCT diagnosed 25 (26.3%) masses to be benign and 70 (73.7%) to be malignant (sensitivity, specificity, positive and negative predictive value and accuracy were 92.8%, 88.0%, 95.5%, 81.4% and 91.5% respectively). The sensitivity and specificity of MDCT in determining local extension was 72.2% and 93.4% respectively. And the sensitivity and specificity of MDCT in determining peritoneal seeding and liver extension was 81.8% and 93% respectively. Estimated stage was significantly agreed with the surgical (Cohen's Kappa (κ) = 0.891) and histopathological findings (κ = 0.858).

**Conclusion:**

MDCT is a highly sensitive and specific diagnostic method in evaluation of adnexal masses and successfully stage the tumor in consistent with surgery and histopathology.

## Background

Malignancies of the female reproductive system are among serious causes of mortality and morbidity, and adnexal cancers are in fifth place among the tumors with the highest mortality in the female population [1]. While the diagnosis may be delayed because of unspecified symptoms, appropriate treatment plan will be achievable with deliberate staging of the tumor and will follow by a better outcome [2]. The presence of an adnexal mass is the leading indications for gynecologic surgery, but the characterization of clinically diagnosed ovarian masses is frequently not possible until surgery and histopathologic examination have been performed. In most institutions the type of surgery (laparotomy vs. laparoscopy) depends on the probability of malignancy, which is based mostly on imaging appearance [3,4].

Putting together with a thorough observation, physical examination and characteristics of the mass will give valuable information about its nature [5,6]. Afterward, several invasive and non-invasive paraclinical evaluations can provide additional information [7,8]. Computed tomography (CT) has been used primarily in patients with ovarian malignancies to reveal the stage of tumor, detect persistent or recurrent disease and demonstrate tumor response to therapeutic approach [9,10].

Computed tomography of abdomen and pelvic can depict the masses as well as probable local or regional invasions. Additionally, it can differentiate gastrointestinal tract, urinary tract and reproductive malignancy from each other using contrast materials. Multi-detector computed tomography (MDCT) makes multiplanar evaluation of pelvic and abdominal structures available as well as two or three dimensional illustrations [11]. Further given details about the extension of the tumors particularly improves the treatment plan and outcome. The newly introduced 64-slice MDCT can provide high quality images of surrounding organs like diaphragm, paracolic gutters and intestine which defines patients who will benefit neo-adjuvant chemotherapy before debulking [12].

Although the diagnostic accuracy of spiral CT and its axial views for nature and extension of the adnexal masses were reported [13], the present study aimed to evaluate the accuracy of 64-multidetector computed tomography in detection, differentiation and staging of adnexal tumors.

## Methods

### Study design

From May 2007 to March 2009 all women with the primary diagnosis of adnexal mass who were referred for further evaluation by MDCT imaging and underwent surgical resection and histopathologic examination, were included in this study. The mass extensions to pelvic, abdominal organs or peritoneum, existence of ascitis and lymph nodes involvement have been evaluated by MDCT, surgical studies and histopathologic examinations. This study has been approved by the Ethics Committee of Tabriz University of Medical Sciences. A written informed consent was obtained from all participants.

### MDCT protocol

All MDCT studies were performed using a 64-multislices MDCT system (Somatom Sensation 64, Siemens medical solutions, Forchheim, Germany) in Tabriz Imam Khomeini Hospital (Parsian Center). Image scanning parameters were as follows: rotation time 1 second, table speed 15.4 mm/rotation, reconstruction interval 0.6 mm at Kernel H20, 120 kV/260 mAs, acquisition time 9s.

MDCT images were obtained from the abdomen and pelvic, covering the area from the diaphragm to the symphysis pubis (craniocaudal). All scans were done with a standard protocol using the triple phase. Precontrast scan of the upper abdomen; arterial phase using the Automatic Bolus Tracking System; portal phase yielded with a delay of 60 s after the arterial one. The contrast medium (Ultravist 370 mg iodine/mL; Schering, Germany) was administered at a dose of 1.5 mL per kg, with a variable flow rate of 3-4 mL per second through the antecubital vein of the right arm.

To facilitate the differentiation of calcified peritoneal implants from bowel loops, 500 ml of water was administrated 30 min prior to the examination. Although it may be difficult to recognize small peritoneal implants and distinguishing them from bowel loops, a careful evaluation of multiplanar reformatted (MPR) images usually enables this differentiation. All patients were fixed during MDCT examination to prevent motion artifacts.

### Image interpretation

The MDCT studies were interpreted at a workstation by an experienced radiologist (M.K.T.; 5 years experience of MDCT and 10 years of CT) using Maximum Intensity Projection (MIP); MPR and volume rendered images (VRI).

### Surgical and Pathological evaluation

An expert surgeon (E. O. S.) reported surgical results and described involvement of pelvis, lymph nodes and peritoneum. An experienced pathologist (A. D.) examined all the resected specimens with no knowledge of the MDCT or surgical findings. The surgical and histopathological findings were considered as a control for the evaluation of MDCT findings in adnexal mass.

### Statistical analysis

Statistical analyses were performed by SPSS software package for windows version 13.0 (SPSS Ins., Chicago, USA). Results are presented as mean ± standard deviation (SD). The sensitivity, specificity, positive and negative predictive value for MDCT were calculated in comparison with surgical and histopathological findings. The Fisher exact test and Pearson correlation tests (Cohen's kappa (κ) values [14]) were used to determine the agreement between MDCT findings and two controls (including surgery and histopathology) findings in stage of adnexal masses. The results were considered significance when the *P *value was less than 0.05.

## Results

**Figure 1:**
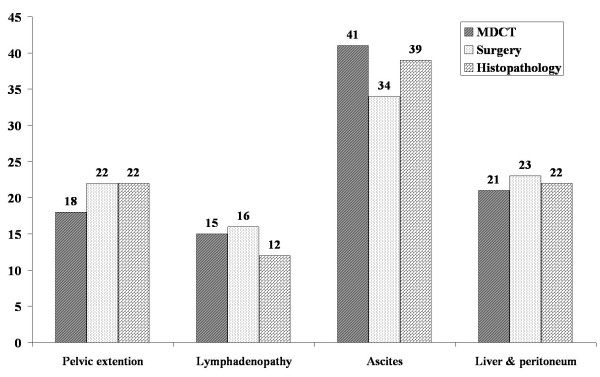
**The frequency of pathologic findings reported by MDCT, surgery and histopathology in patients with adnexal mass**.

**Figure 2:**
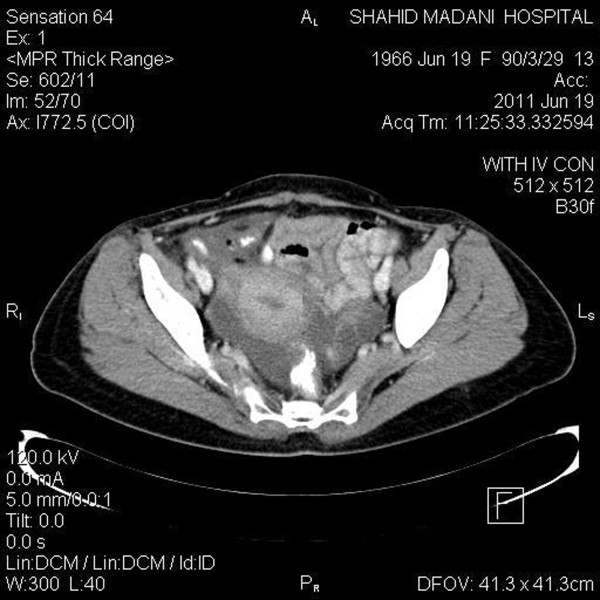
**Left Adnexal mass - Axial view**.

During the study period 95 women with a primary diagnosis of ovarian mass (mean age, 48.63 ± 13.93 years) were included in the study. The frequency of pathologic findings reported by MDCT, surgery and histopathology are demonstrated in Figure 1. MDCT diagnosed 25 (26.3%) masses to be benign and 70 (73.7%) masses to be malignant. The sensitivity, specificity, positive and negative predictive value and accuracy of MDCT for diagnosing a malignant mass was 92.8%, 88.0%, 95.5%, 81.4% and 91.5% respectively comparing to histopathological evaluation of the specimens (Figure 2).

**Figure 3:**
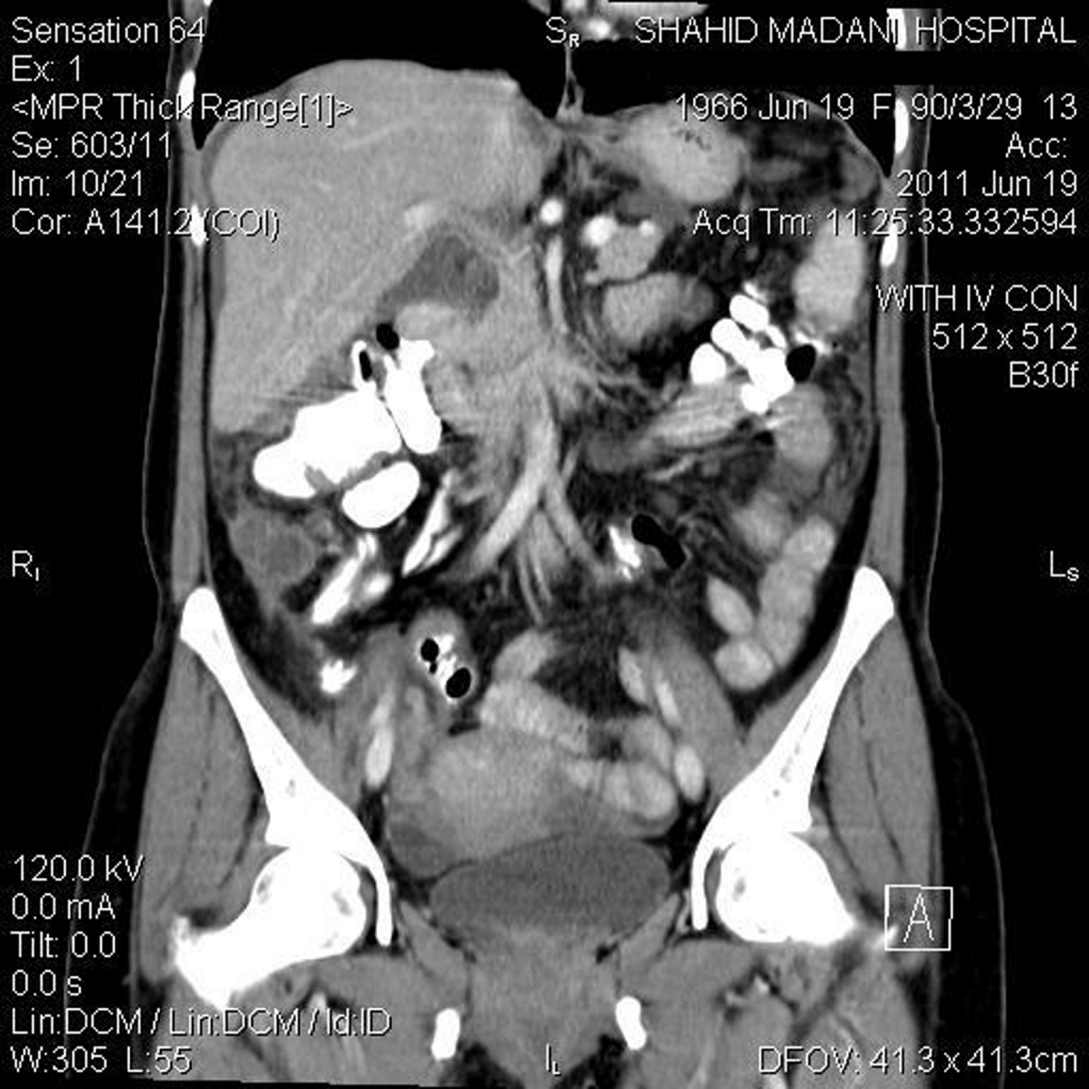
**Suprapubic peritoneum involvement - Coronal view**.

**Figure 4:**
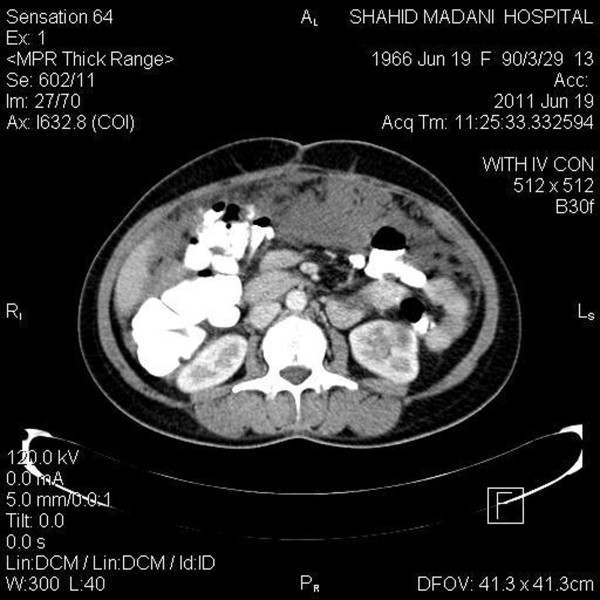
**Omental seeding - Transverse view**.

Table 1 presents the diagnostic performance of MDCT for detecting involvement of organs (pelvis, lymph nodes and peritoneum) in comparison to surgical and histopathological findings. In comparison with surgical and histopathologic findings, accuracy of MDCT was 90.7% and 90.7% for detecting pelvic involvement, 95.35% and 92.3% for detecting lymph nodes distribution, and 89.2% and 89.2% for peritoneal involvement (Figure 3 &4). In addition, sensitivity of MDCT was 91.1% for detecting ascites (compared to report of the surgical findings), while specificity of it for detecting a malignant cell inside it was 43.3% (compared to histopathologic findings).

**Table 1:** The diagnostic performance of MDCT for detecting involvement of other organs in patients with an adnexal mass

	MDCT compared to:	Sensitivity	Specificity	PPV	NPV	Accuracy
Pelvis	Surgery	80.9%	95.4%	89.4%	91.3%	90.7%
	
	Histopathology	72.2%	93.4%	84.2%	87.75	90.7%

Lymph nodes	Surgery	81.2%	100%	100%	94.2%	95.35
	
	Histopathology	83.3%	94.3%	76.9%	96.1%	92.3%

Peritoneum	Surgery	79.1%	95.1%	90.45	88.65	89.2%
	
	Histopathology	81.8%	93%	85.7%	90.9%	89.2%

PPV: Positive predictive value, NPV: Negative predictive value. MDCT: multi-detector computed tomography.

The disagreement in staging of the tumor between MDCT and surgery was found in 12 cases (table 2). Adnexal masses were overstaged in three patients and understaged in nine patients. Such disagreement observed in 10 cases in comparison to histopathologic findings; three patients were overstaged and seven understaged. However, there were significant agreements between MDCT and surgical findings (κ = 0.891) and between MDCT and histopathologic findings (κ = 0.858).

**Table 2:** Staging of the tumors and agreement between methods

	MDCT (n)	Surgery (n)	Histopathology (n)
I	30	27	28

II	6	7	6

III	24	26	26

IV	5	5	5

MDCT: multi-detector computed tomography.

## Discussion

The results of the present study describe the significant agreement between MDCT, surgery and histopathology in determining stages of adnexal masses. Also, it has been demonstrated that MDCT have high efficacy and accuracy in defining the nature of a pelvic mass and detecting extension of malignant tumors which could be very useful in planning of treatment.

Adnexal masses are usually detected by clinical examination or sonography. Once an ovarian mass is detected, determination of a degree of suspicion for malignancy is important and is based largely on imaging appearance. Ultrasound (US) is considered the primary imaging modality for the assessment and characterization of adnexal masses. But, although the reported sensitivities of the technique are high (85-100%), the specificities are variable (50-100%) [15,16]. Several studies have suggested that CT can play an important role in characterizing ovarian masses, emphasizing the comparability of CT to other imaging modalities such as magnetic resonance imaging (MRI) or US [17,18]. Sensitivity and specificity of contrast-enhanced helical CT is reported to be 88-90% and 88-89% (respectively) for distinguishing malignant and benign adnexal masses [19]. The rates are 89-91% (sensitivity) and 88-93% (specificity) when using MRI [20,21], while different kinds of ultrasonography have a sensitivity of 35-99% with lower rates of specificity [22,23].

Tsili et al. reported the sensitivity of the16-slice MDCT to be 90% and accuracy of 89.1% for detecting malignant tumors in patients with an adnexal mass [24]. However, higher sensitivity (90.5%) and accuracy (92.9%) were reported by the same MDCT imaging method later [25]. Improved results in the present study might be due to thinner slices (64-multi-slices) of MDCT which got enhanced by reconstructed images.

The sensitivity, specificity, positive and negative predictive value and accuracy of CT has some potential limitations which are the topic of today researches to be improved. The sensitivity of CT for detecting peritoneal metastasis is reported about 85-93%, while it decreases to 20-25% when the metastasis is lesser than 1 cm in diameter [26]. This may be minimized by thinner slices, loss of artifacts due to partial volume effect and multiplanar reformatting which makes it possible to evaluate bending planes as well [27].

Results of MDCT imaging were compatible with histopathological findings in 84.6%. When there was a different MDCT mostly under-estimated the stage of the tumor. Accuracy of MDCT was higher in advanced stages compared to earlier stages (I and II). This maybe explained by high capability of MDCT to illustrate peritoneal seeding and involvement of abdominal visceral organs. Similar results have been reported by Tsili et al. about the compatibility between results of MDCT and histopathological evaluations [24] which is reported to be 85%. The main difference is that the majority of our study population had malignant tumors.

This study concludes that despite the possibilities of overstaging and understaging the adnexal masses, MDCT provides accurate information on detection, differentiation and staging of adnexal tumors and allows planning therapeutic approach.

## List of abbreviations

CT: Computed Tomography; MDCT: Multi-Detector Computed Tomography; MPR: Multiplanar Reformatted; MIP: Maximum Intensity Projection; VRI: Volume Rendered Images; US: Ultrasound; MRI: Magnetic Resonance Imaging.

## Competing interests

The authors declare that they have no competing interests.

## Authors' contributions

All the authors in this manuscript have read and approve the final manuscript. MGS: Conception and design, and manuscript writing. MKT: The MDCT studies and manuscript writing. EOS: Surgical results. AD: Pathological examinations. AA: Data analysis and manuscript writing.
